# Learning curve in image-based robotic assisted total knee arthroplasty: a MAKO-robot experience

**DOI:** 10.1007/s00590-026-04803-0

**Published:** 2026-06-08

**Authors:** Ferdinando Granata, Francesco Bosco, Claudio Domenico Cobisi, Federico Nasca, Riccardo Giai Via, Mariazzurra Carlino, Jonathan M. Vigdorchik, Carmelo Burgio

**Affiliations:** 1 Ospedale G.F. Ingrassia, Palermo, Italy; 2 University of Palermo, Palermo, Italy; 3 University of Turin, Turin, Italy; 4 University of Bologna, Bologna, Italy; 5 Hospital for Special Surgery, New York, USA

**Keywords:** Robotic-assisted total knee arthroplasty, MAKO, Learning curve, CUSUM analysis, Surgical workflow

## Abstract

**Purpose:**

Robotic-assisted total knee arthroplasty (RA-TKA) is increasingly used to improve implant positioning, soft-tissue balance, and procedural reproducibility. Yet, little is known about how different components of the operation independently contribute to the overall learning curve. This study aimed to characterize the learning curve of MAKO-assisted TKA by separately evaluating these components and assessing their differential impact on operative workflow.

**Methods:**

A retrospective observational study included 92 consecutive patients who underwent image-based RA-TKA (MAKO, Stryker) for primary knee osteoarthritis. All procedures were performed by a single experienced arthroplasty surgeon with no prior robotic or computer-assisted surgery background. Cumulative sum (CUSUM) analysis with piecewise linear regression was applied to total surgical time, pin placement time, and composite robotic workflow time to identify inflection points and define learning curve phases.

**Results:**

Piecewise regression of the CUSUM plot for total surgical time revealed two breakpoints at cases 11 and 51, defining three phases of the learning curve: (1) initial learning (cases 1–11), (2) competence (cases 12–51), and (3) optimized performance (cases 52–90). Mean surgical time was 68.9 ± 20.1 min, stabilizing around 65 min after 50 cases. Along with total surgical time, the initial learning phase ended around cases 10–11 for both robotic workflow and pin placement. However, subsequent performance patterns differed: pin placement reached optimized performance by case 52 (mean 8.4 ± 4.3 min), whereas robotic workflow time improved more gradually, without clear stabilization until the end of the series (mean 37.8 ± 10.8 min).

**Conclusions:**

RA-TKA with the MAKO system follows a structured learning curve with early achievement of proficiency after 11 cases. Total surgical time and pin placement reached optimized performance by mid-series, whereas robotic workflow tasks required a longer consolidation period, likely influenced by patient-specific anatomical variability. These findings support RA-TKA as a safe and effective tool, offering rapid surgeon adaptation.

**Level of evidence:**

IV.

**Supplementary Information:**

The online version contains supplementary material available at 10.1007/s00590-026-04803-0.

## Introduction

Robotic-assisted total knee arthroplasty (RA-TKA) has rapidly expanded over the past decade, driven by the need to improve surgical accuracy, optimize limb alignment, and achieve more reproducible soft-tissue balance—factors long associated with postoperative dissatisfaction and early failure following conventional TKA [1, 2]. Compared with manual instrumentation, RA-TKA enables greater precision in bone resection and alignment, with the potential to reduce outliers and enhance the reproducibility of implant positioning, while also supporting soft-tissue preservation [3–5].

Robotic platforms can be broadly classified into image-based and imageless systems. Although previous studies have not demonstrated clinically meaningful differences in alignment accuracy between these technologies [6], they differ substantially in their approach to anatomical data acquisition. Image-based platforms rely on preoperative CT imaging to generate a detailed, patient-specific 3D model, enabling highly accurate preoperative planning. In contrast, imageless systems reconstruct a virtual bone model intraoperatively through surface registration. Image-based systems may offer slightly superior accuracy but at the cost of reduced cost-effectiveness and additional radiation exposure [6–8].

Among image-based solutions, the MAKO robotic platform (Stryker, USA) is one of the most widely adopted worldwide. Its use has been associated with improved implant positioning, reduced postoperative pain, and superior early functional results compared with conventional TKA, supporting its role in delivering a patient-specific, highly reproducible surgical approach [9]. The precision enabled by MAKO has also facilitated the adoption of functional alignment (FA), an alignment philosophy positioned between mechanical and kinematic alignment that aims to restore individualized knee biomechanics while respecting each patient’s native soft-tissue envelope [10, 11].

As robotic technology becomes increasingly integrated into routine arthroplasty practice, defining its learning curve is essential for understanding operative efficiency, resource utilization, and safe implementation. Therefore, this study aimed to characterize this learning curve by evaluating how key intraoperative time components evolved across consecutive procedures. Specifically, we assessed changes in total surgical duration, the time required for registration pin placement, and the time dedicated to the robotic workflow, including registration, intraoperative planning, and execution of bone resections, as experience with the system increased.

## Materials and methods

### Study design

A retrospective observational study was conducted on consecutive patients who underwent RA-TKA using the MAKO system (Stryker, Kalamazoo, MI, USA) between September 2023 and June 2025 at a single referral center. A total of 92 procedures were included. All procedures were performed by the same senior surgeon (F.G.), a high-volume arthroplasty surgeon performing approximately 100 primary total knee arthroplasties per year but who had no prior exposure to computer-assisted or RA-TKA at the beginning of the study period. Throughout all cases, the robotic system functioned without registration failures, workflow disruptions, or technical malfunctions. A dedicated MAKO clinical specialist was present during all procedures, in accordance with standard institutional practice, providing technical support for system setup and workflow management without direct involvement in surgical decision-making.

### Inclusion and exclusion criteria

Eligible patients were those diagnosed with primary knee osteoarthritis classified as Kellgren–Lawrence grade III or IV and treated with primary RA-TKA using the MAKO robotic platform. Patients were excluded if they required revision surgery, conversion from unicompartmental to TKA, or if they presented with inflammatory arthropathy or post-traumatic arthritis with retained hardware.

### Surgical procedure

All procedures were performed under spinal or general anesthesia with the patient positioned supine. A lateral thigh post and foot bolster were used to permit controlled knee flexion and extension, and a pneumatic tourniquet was routinely applied. Intravenous tranexamic acid was administered per institutional protocol. A medial parapatellar arthrotomy was utilized for all cases, most commonly through a mid-vastus approach to provide direct joint exposure.

Proximal tibial and distal femoral registration pins were placed, and optical tracking arrays were mounted securely. The MAKO robotic arm and console were positioned adjacent to the operative field in accordance with system guidelines. Bone registration was then performed to identify key anatomical landmarks and establish the mechanical axes of the femur and tibia. Once registration accuracy was verified, the planning interface was used to adjust coronal and sagittal alignment parameters, joint-line level, and component orientation. Virtual component positioning was assessed dynamically throughout knee range of motion.

The robotic arm subsequently guided execution of the planned femoral and tibial resections using haptic boundaries to ensure controlled, precise bone preparation. After all cuts were completed, trial components were inserted, and overall limb alignment, implant fit, range of motion, and patellar tracking were evaluated. Once the reconstruction was deemed satisfactory, definitive components were implanted using standard cementation techniques. The procedure concluded with thorough irrigation, meticulous hemostasis, and layered closure of the arthrotomy and skin.

### Data collection

For each consecutive procedure, granular intraoperative timing data were prospectively recorded to characterize the surgeon’s progression along the robotic learning curve. Three core temporal metrics were analyzed. Total surgical time was defined as the cut-to-cut interval from initial skin incision to final wound closure. Pin placement time represented the duration required to position the cortical pins, mount the optical arrays, and confirm adequate fixation. Robotic workflow time captured all elements unique to the robotic-assisted portion of the operation, including anatomic registration, verification and calibration of the system, intraoperative plan refinement, execution of robot-guided bone resections, and final confirmation of component alignment. These metrics were extracted from operative records and analyzed chronologically to evaluate performance progression across the series.

### Statistical analysis

Descriptive statistics were generated for all variables. Continuous variables were summarized as mean ± standard deviation, whereas categorical variables were reported as absolute frequencies and percentages. The learning curve for robotic-assisted TKA was evaluated using cumulative sum (CUSUM) analysis of total surgical time, pin placement time, and robotic workflow time, following previously described methods for RA-TKA learning-curve assessment [14, 15]. CUSUM analysis characterizes changes in operative performance over sequential cases by quantifying the running deviation of each operative time from the overall mean. Positive slopes on the CUSUM plot indicate performance slower than the cohort mean, whereas negative slopes indicate improving efficiency with times below the mean. To identify distinct phases of the learning curve, piecewise linear regression models were fitted to each CUSUM curve. All statistical analyses were performed using RStudio (version 3.3.2; R Foundation for Statistical Computing, Vienna, Austria).

## Results

A total of 92 RA-TKA procedures were included, comprising 37 males (40.2%) and 55 females (59.8%) with a mean age of 69.92 ± 8.02 years (range 43–84). Right-sided procedures accounted for 56.6% of cases, while left-sided procedures accounted for 43.4%.

**Fig. 1:**
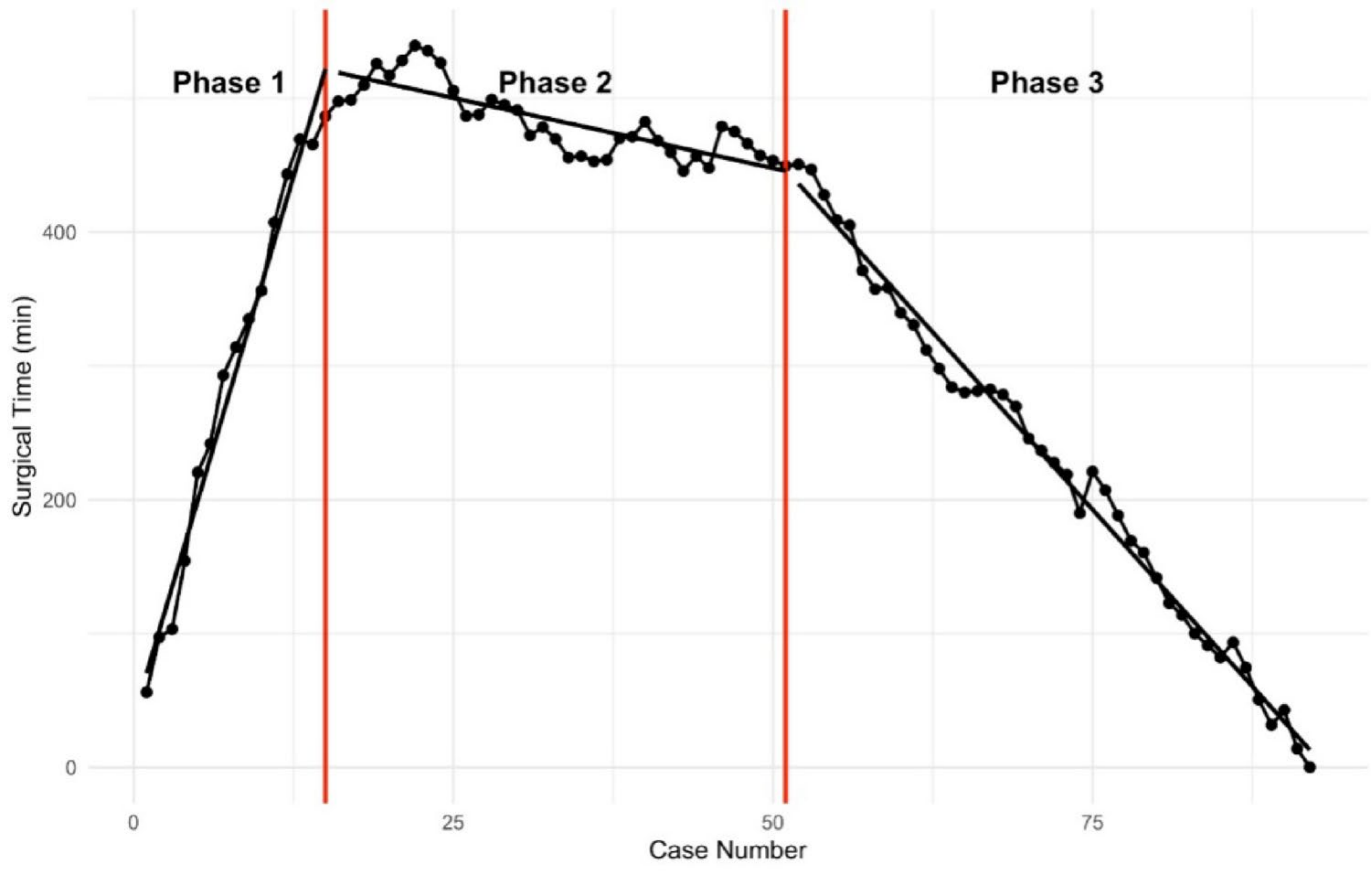
CUSUM learning-curve analysis for total surgical time. Black dots represent the cumulative deviation of robotic workflow time for each sequential case. The solid black line shows the fitted piecewise regression model used to detect changes in performance trends. The red vertical line marks the breakpoint identified by the model

**Fig. 2:**
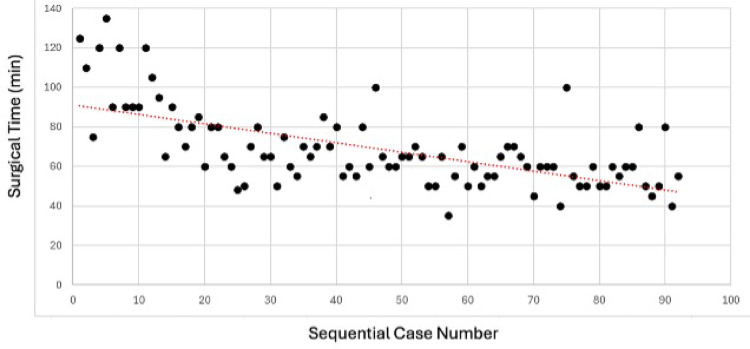
Scatterplot of Total Surgical Time Across Sequential RA-TKA Cases. Black dots represent the robotic workflow time for each individual case. The red dotted line shows the fitted linear regression trend

Analysis of the CUSUM curve for total surgical time identified two statistical significant inflection points, delineating three distinct phases of the learning curve (Fig. 1). The first breakpoint at case 11 marked the end of the initial learning phase, during which operative times were consistently above the cohort mean. The second breakpoint at case 51 signaled the transition from the competence (plateau) phase into the optimized performance phase. Accordingly, Phase 1 (cases 1–11) demonstrated an upward cumulative trend consistent with early adaptation; Phase 2 (cases 12–51) exhibited operative times that approximate the cohort mean but still demonstrated variability; and Phase 3 (cases 52–90) showed a downward trajectory, indicating operative times became consistently faster than the average. The corresponding scatterplot demonstrated a clear decline in total surgical time across sequential cases, with durations stabilizing around 65 min after 50 cases, as illustrated by the fitted linear regression trendline (Fig. 2). Overall, the mean surgical time across the cohort was 68.9 ± 20.1 min.

**Fig. 3:**
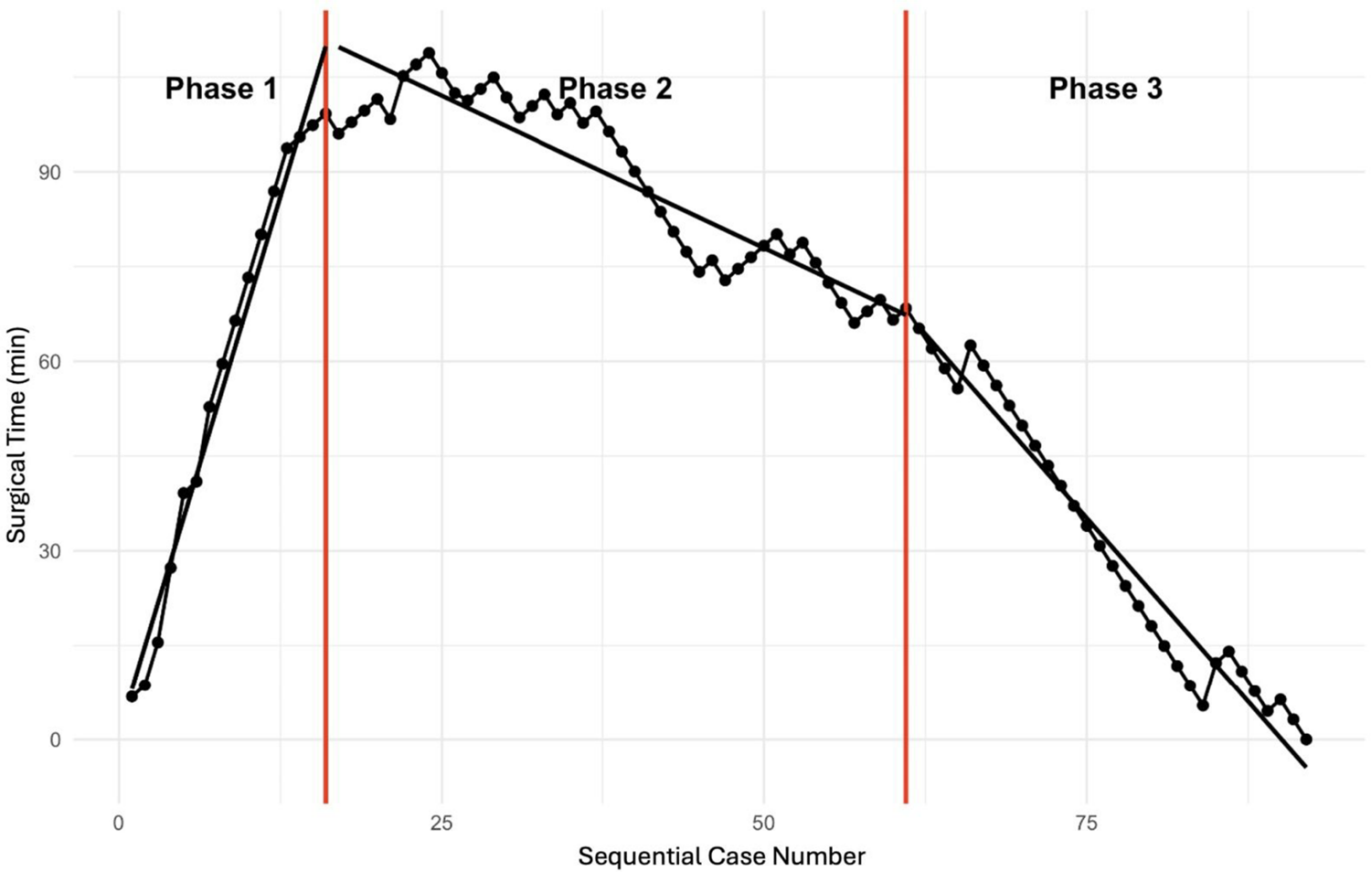
CUSUM learning-curve analysis for pin placement. Black dots represent the cumulative deviation of robotic workflow time for each sequential case. The solid black line shows the fitted piecewise regression model used to detect changes in performance trends. The red vertical line marks the breakpoint identified by the model

**Fig. 4:**
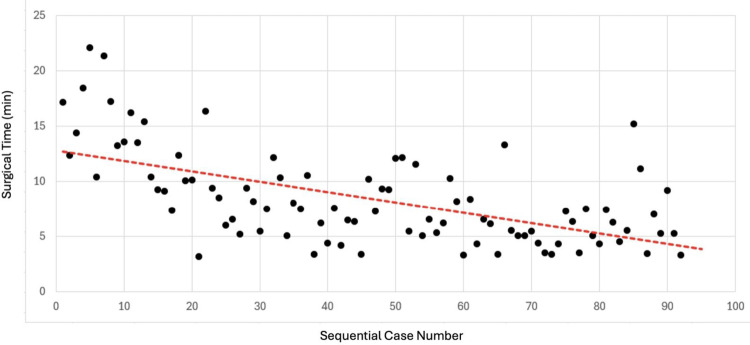
Scatterplot of Pin Placement Time Across Sequential RA-TKA Cases. Black dots represent the robotic workflow time for each individual case. The red dotted line shows the fitted linear regression trend

Pin placement time demonstrated a similar statistically significant three-phase progression (Fig. 3). Phase 1 (cases 1–11) demonstrated a rising cumulative trend consistent with early adaptation to robotic array placement. Phase 2 (cases 12–51) showed a relative plateau, with times closer to the mean but still variable. Phase 3 (cases 52–90) exhibited a clear downward trajectory, reflecting faster and more consistent pin placement as the workflow became fully integrated. Sequential case plotting revealed a steady decline from approximately 12–15 min during initial procedures to ≤ 5 min in later cases (Fig. 4). The mean pin placement time for the entire cohort was 8.4 ± 4.3 min.

**Fig. 5:**
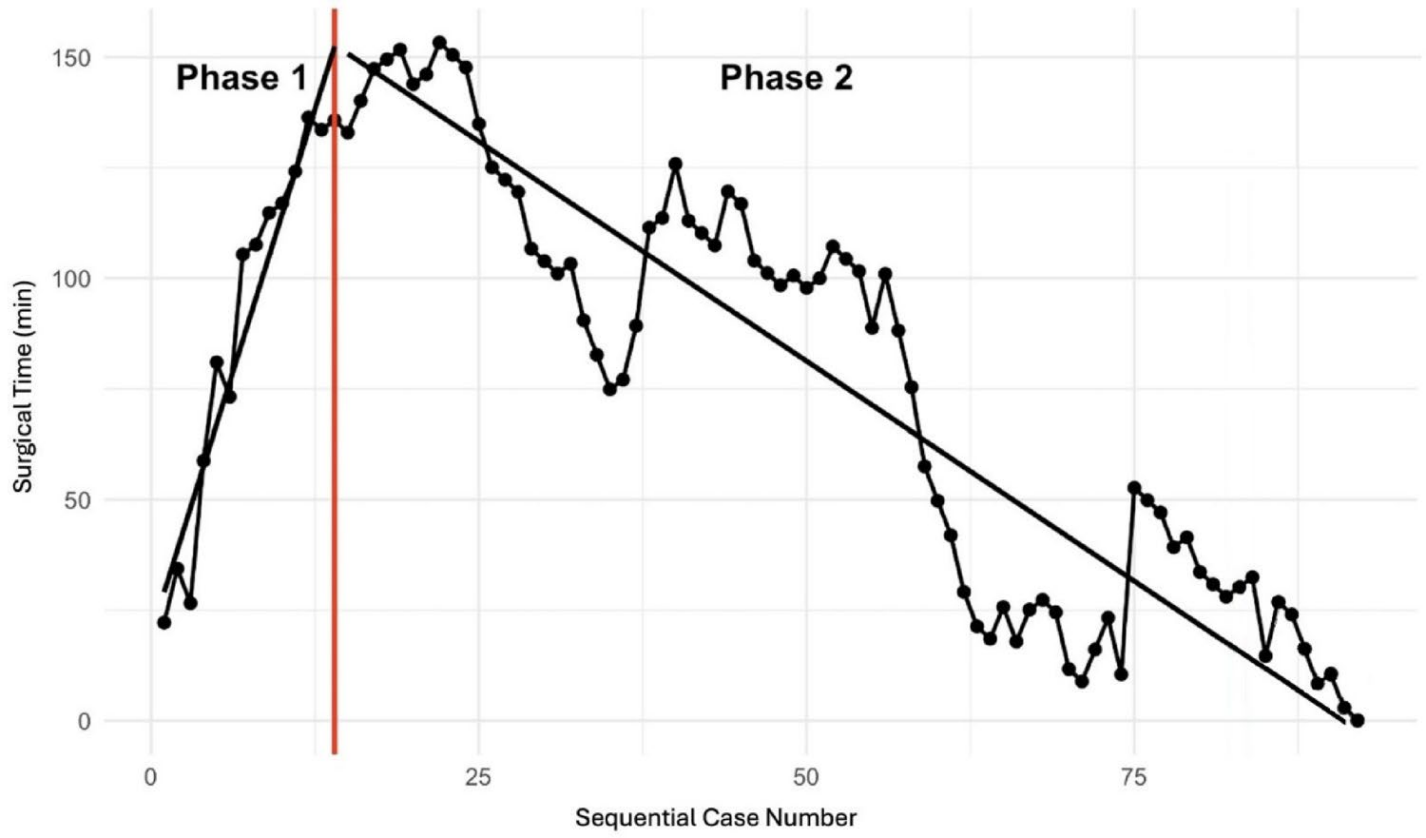
CUSUM learning-curve analysis for robotic workflow time. Black dots represent the cumulative deviation of robotic workflow time for each sequential case. The solid black line shows the fitted piecewise regression model used to detect changes in performance trends. The red vertical line marks the breakpoint identified by the model

For robotic workflow time, CUSUM analysis identified a single significant inflection point, delineating two distinct phases of the learning curve (Fig. 5). The breakpoint occurred at case 11, marking the end of the initial learning phase, during which workflow times were consistently above the cohort mean. Beyond this point, Phase 2 showed an overall downward cumulative trend with variable improvement, indicating enhanced familiarity with the robotic steps. However, no distinct optimized-performance phase (Phase 3) could be identified, indicating that workflow optimization progressed steadily rather than in discrete stages. Scatterplot analysis corroborated a modest, progressive reduction over time, with workflow durations generally ranging from 30 to 50 min throughout the series (Supplemental Fig. 1). The mean robotic workflow time was 37.8 ± 10.8 min.

## Discussion

In this consecutive series of 92 image-based MAKO RA-TKA procedures performed by a surgeon with no prior robotic experience, we identified distinct learning patterns across surgical domains. Total surgical time and pin placement time demonstrated clear three-phase learning curves, with the initial learning phase completed after approximately 11 cases and optimized performance achieved by around case 52. In contrast, robotic workflow time followed a two-phase pattern: although efficiency improved after the initial learning phase, no discrete optimized-performance phase emerged, reflecting continued variability and a more gradual refinement of robotic tasks. Overall, operative efficiency improved consistently across the series, indicating that surgeons can achieve early proficiency while continuing to refine workflow components over a broader range of cases.

Our findings align closely with previously published MAKO-specific literature. Kayani et al. reported that operative time plateaued after approximately 20 cases, with no learning effect observed for implant alignment or soft-tissue balancing, which remained consistently accurate across all cases [12–15]. Previous systematic reviews similarly estimated the learning curve for MAKO-assisted TKA at 15–25 procedures depending on the metric analyzed [14, 15]. These results collectively reinforce that, although MAKO-assisted workflows require initial familiarization, precision-related advantages are present from the earliest stages of adoption.

Comparison with other robotic platforms highlights variability in learning dynamics across systems. For the ROSA platform, several studies have described a relatively short learning curve of 6–14 cases, accompanied by a substantial reduction in operative time during the transition from early learning to competence [16]. Conversely, other analyses suggest that up to 70 procedures may be required before operative times approximate those of conventional TKA, reflecting diverse institutional workflows and differences in prior exposure to navigation technologies [17].

The Navio system exhibits a longer learning curve, with proficiency reported after 25–40 cases and further improvements extending beyond 80 procedures in some series [18, 19]. In contrast, Cori appears to require fewer cases, with proficiency documented after as few as six procedures, although early complications—including over-resection and pin-related issues—were reported during the adjustment phase [12]. Emerging data on the Velys system suggest high accuracy from the earliest cases with progressive reductions in operative time as experience increases [12, 20].

These platform-specific variations are reflected in recent meta-analyses involving more than 9,000 RA-TKAs, which estimate a median learning curve of approximately 17 cases (IQR 9–27) and document a mean reduction of 14–17 min from early to competent phases [21]. Importantly, across robotic systems, the precision of component positioning and limb alignment is typically consistent even during the initial learning period, with operative time representing the primary parameter influenced by surgeon experience [9, 13]. Taken together, our data position the MAKO platform within the mid-range of robotic learning curves, longer than Cori and ROSA but shorter than Navio [15, 18–21].

Importantly, the differing maturation profiles of total surgical time, pin placement, and robotic workflow underscore that not all components of RA-TKA progress at the same pace. While the initial learning phase is surpassed relatively early, reflecting rapid familiarization with the core robotic steps, complete consistency in robotic workflow tasks develops more gradually. This slower consolidation likely reflects the greater sensitivity of these tasks to patient-specific variability in anatomy and deformity, which influences the time needed for registration, planning refinements, bone preparation, and haptic execution. These observations highlight that, beyond surgeon skill acquisition, structured onboarding, standardized operating room workflows, and consistent team involvement play essential roles in supporting efficient and safe adoption of RA-TKA [22].

A significant strength of this study is the inclusion of a consecutive series of RA-TKA procedures performed by a single surgeon with extensive experience in conventional TKA but no prior exposure to robotic platforms. This allows clear identification of the actual early learning curve, free from confounding from prior robotic expertise. Additionally, the use of CUSUM analysis and piecewise regression provides a robust statistical approach to characterize learning phases and inflection points.

### Limitations

However, several limitations must be acknowledged. First, this is a single-surgeon, single-center study, which may limit generalizability to institutions with different workflows, staff expertise, or training pathways. Second, only intraoperative time metrics were analyzed; evaluation of clinical outcomes, complications, or implant positioning could further contextualize the learning curve. Third, the absence of a comparison group (e.g., conventional TKA or other robotic platforms) prevents direct benchmarking against alternative techniques. Fourth, although workflow times were carefully recorded, subtle variations in team experience or operating room logistics may have influenced the observed trends.

Another potential confounding factor may be represented by the concurrent introduction and adoption of a new alignment philosophy (FA) in surgeon’s experience. Additionally, as all procedures were performed by a single experienced surgeon, the findings may not be fully generalizable to surgeons with different levels of expertise or varying familiarity with robotic systems. The technical demands of robotic-assisted TKA, together with inter-patient anatomical variability, may further influence the reproducibility of the observed learning curve across different clinical settings. Therefore, caution should be exercised when extrapolating these results to broader surgical populations. Moreover, a direct comparison with the surgeon’s baseline operative time for conventional TKA was not possible, as these data were not systematically recorded prior to the introduction of robotic-assisted techniques. This limits the ability to fully contextualize the observed operative times relative to pre-existing surgical performance.

## Conclusion

This study demonstrates that MAKO-assisted TKA follows a distinct learning curve characterized by an early adaptation period, a competence plateau, and a final phase of optimized performance. The initial learning phase was surpassed after approximately 11 cases, for all the parameters investigated, reflecting rapid acquisition of RA-TKA technical skills. However, while total surgical time and pin placement time stabilized by around case 52, indicating early mastering, the robotic workflow progressed more gradually, likely influenced by patient-specific anatomical variability and planning complexity. Given the absence of clinical outcome and complication data, these findings should be interpreted primarily in terms of procedural performance and workflow adaptation rather than clinical safety or effectiveness.

## Supplementary Information

Below is the link to the electronic supplementary material.

Supplementary Material 1: Supplemental Figure 1. Scatterplot of Robotic Workflow Time Across Sequential Cases. Black dots represent the robotic workflow time for each individual case. The red dotted line shows the fitted linear regression trend.

## Data Availability

The authors declare that data supporting the findings of this study are available within the articles included in this retrospective study.
