# Supplementary figures and images for: Learning curve in image-based robotic assisted total knee arthroplasty: a MAKO-robot experience
Source: Eur J Orthop Surg Traumatol. 2026 Jun 8;36(1):217. doi: 10.1007/s00590-026-04803-0 (PMC13246530; doi:10.1007/s00590-026-04803-0)

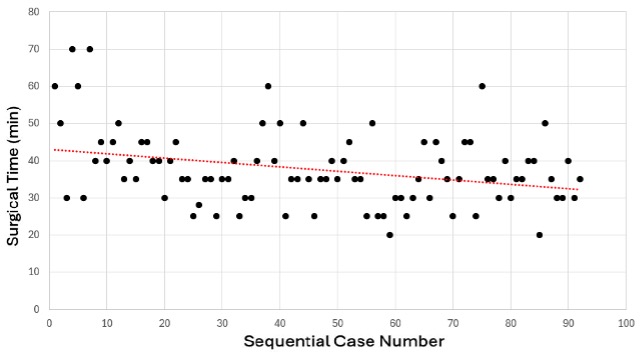

Supplement: Supplementary file 1 — Supplementary Material 1: Supplemental Figure 1. Scatterplot of Robotic Workflow Time Across Sequential Cases. Black dots represent the robotic workflow time for each individual case. The red dotted line shows the fitted linear regression trend. [file 590_2026_4803_MOESM1_ESM.jpg]
